# DNA methyltransferase expression (DNMT1, DNMT3a and DNMT3b) as a potential biomarker for anti-VEGF diabetic macular edema response

**DOI:** 10.1177/11206721231171623

**Published:** 2023-04-20

**Authors:** Pedro Camacho, Edna Ribeiro, Bruno Pereira, Teresa Varandas, João Nascimento, José Henriques, Marco Dutra-Medeiros, Mariana Delgadinho, Ketlyn Oliveira, Carina Silva, Miguel Brito

**Affiliations:** 1 H&TRC- Health & Technology Research Center, ESTeSL- Escola Superior de Tecnologia da Saúde, Instituto Politécnico de Lisboa, Lisbon, Portugal; 2 Ophtalmology Institute Dr. Gama Pinto, Lisbon, Portugal; 3 iNOVA4Health, NOVA Medical School, Faculdade de Ciências Médicas, NMS, FCM, Universidade NOVA de Lisboa, Lisbon, Portugal; 4 Retina Institute of Lisbon, Lisbon, Portugal; 5 Portuguese Diabetes Association (APDP), Lisbon, Portugal; 6 Beatriz Ângelo Hospital, Lisbon, Portugal; 7 Central Lisbon Hospital Center, Lisbon, Portugal

**Keywords:** Diabetic retinopathy, diabetes mellitus, macular edema, DNA methylation, epigenetics

## Abstract

**Purpose:**

DNA methylation is involved in Diabetic Retinopathy progression showing a metabolic memory mechanism. However, the association of DNA methyltransferase with diabetic macular edema is still unknown. We aimed to describe the differences in DNA methyltransferase gene expression in patients with different diabetic macular edema responses.

**Methods:**

A total of 27 diabetic patients, aged 59–90 years, were prospectively enrolled in this cross-sectional study. The participants were classified into control group (CG, n = 11), diabetic macular edema responders (rDME, n = 9) and non-responder diabetic macular edema (nrDME, n = 7) after anti-vascular endothelial growth factor (anti-VEGF) treatment. Only cases with a complete ophthalmological examination, digital 133° color fundus, and SD-OCT assessments were used. After RNA extraction and first-strand cDNA synthesis, quantitative real-time PCR was performed with specific primers on the CFX Connect™ Real-Time PCR Detection System to assess differential transcriptional expression patterns.

**Results:**

The DNMT1 gene showed a positive correlation (r = 0.617; p = 0.043) with Best Corrected Visual Acuity (BCVA) in CG, a positive correlation (r = 0.917; p = 0.010) with HbA1c in nrDME and a negative correlation (r = −0.659; p = 0.049) with GCL-IPL thickness in rDME. DNMT3A gene showed a positive correlation (r = −0.890; p = 0.001) with Sub-foveal Choroidal thickness in rDME whereas DNMT3b gene showed a negative correlation (r = −0.815; p = 0.007) with HbA1c and RNFL (r = −0.664; p = 0.026) in CG.

**Conclusions:**

Patients with similar metabolic profile risk factors showed associated DNA methyltransferase transcriptional expression patterns differences fitting with the anti-VEGF diabetic macular edema response. Further studies are needed to clarify if these results (1) reflect disease evolution, (2) translate the therapeutic impact, (3) or can help to predict the therapeutic resistance profile.

## Introduction

With estimates of around 750 million by 2030, Diabetes Mellitus (DM) is one of the chronic pathologies with the greatest social and economic impact that continues to challenge the public health policies.^1^

Chronic and persistent hyperglycemia contributes to multiple interlinked metabolic pathways at the origin of ocular manifestations.^2^ However, metabolic control and disease duration are insufficient to explain the variability of disease progression.^3^ Despite the usefulness of these biomarkers in assessing the risk of developing Diabetic Retinopathy (DR), they do not explain why some good metabolic control patients develop Diabetic macular edema (DME) and others with poor metabolic control patients do not.^4^

The abnormal retinal fluid accumulation in DME, the main cause of visual impairment in DR,^1^ is one important challenge in ophthalmology because even with the rigorous treatment schedules and close metabolic control near to 40% of patients with DME are not responsive.^5–7^

Angiogenesis and inflammation have been shown to have an important role in DME pathogenesis,^8^ however real-life data shows that non-responder DME (nrDME) has an inconsistent response and probably other mechanisms must be involved.^9^

Since traditional biomarkers are insufficient to explain the progression of the disease^3^ and the genetic risk factors identified by GWASs do not explain more than 10% of Type 2 DM heritability,^10^ the interest in epigenetics research is growing. And due to the reversible characteristics of epigenetic mechanisms, which provide new therapeutic targets for retinal diseases,^11,12^ this trend has been reinforced.

DNA methylation is a universal mechanism that could be associated with important ocular diseases such as age-related macular degeneration,^13,14^ Diabetic Retinopathy^11,12^ or Glaucoma.^15^ More recently, it was demonstrated that DNA methylation is involved in DM early stages showing a metabolic memory mechanism.^16^ Also, the relationship between global DNA methylation and DR progression,^17,18^ or the expression of oxidative stress and inflammation (IL-1β, IL8 and IL10) genes with the therapeutic responses were described.^12^

Nevertheless, despite the increasing application of DM epigenetics, there is still no data on its relationship with DME. With a three-arm cross-sectional approach (Control Group, responder DME Group, and non-responder DME Group), this study aims to explore for the first time DNA methylation as a biomarker for the treatment of nrDME and to describe the differences in DNA methyltransferase expression (DNMT1, DNMT3A e DNMT3B) in blood lymphocytes, associated with de novo and maintenance methylation.

## Materials and methods

### Study design

The study was carried out at Lisbon School of Health Technology (ESTeSL), Retina Institute of Lisbon (IRL) and Portuguese Diabetes Association (APDP) after approval from each Institutional Ethical Review Board and according to the principles of the Declaration of Helsinki. After a complete explanation to each study participant of the purposes and contribution of the study, free written informed consent was obtained.

Using a convenience non-probable sampling, a total of 27 diabetic patients were prospectively enrolled in this exploratory cross-sectional study at the normal IRL and APDP consultation practices. With a three-arm approach the participants were classified into control group (CG), diabetic macular edema responders (rDME) and non-responder diabetic macular edema (nrDME) after anti-vascular endothelial growth factor (anti-VEGF) treatment. The DR classification was performed by two independent experienced graders (IRL and APDP) according to the Early Treatment Diabetic Retinopathy Study (ETDRS) classification and the eye with the highest central retinal thickness (CRT) and treatment duration was chosen.

### Inclusion criteria and group definition

Due to the difficult in identifying responder and/or non-responder DME a more conservative criteria were chosen. Based on Diabetic Retinopathy Clinical Research network (DRCR.net) Protocol I study and others groups only patients with at least three monthly treatments with anti-VEGF with at least 6 months of follow-up were considered.^5,12,19,20^

- rDME Group: Participants with DME (baseline central subfield thickness ≥305 μm in women and ≥320 μm in men), with a reduction in thickness >10% CRT on SD-OCT^12^ and study eye with early Best Corrected Visual Acuity (BCVA) response (≥5 letters)^19^;
- nrDME Group: Participants with pDME (baseline central subfield thickness ≥305 μm in women and ≥320 μm in men), with a stable/worsening/improvement <10% microns thickness on SD-OCT^12^ at least 180 days after treatment and suboptimal early BCVA response(<5 letters);
- Control Group: Age-matched participants Diabetic without DR. The Control Group (CG) were participants who used the IRL for general ophthalmology consultation.

*Exclusion criteria:* uncontrolled systemic disease, intraocular pressure (IOP) >21 mmHg, or presence of age-related macular degeneration, glaucoma, or vitreomacular disease in the study eye, and high ametropia (spherical equivalent greater than −6 or +2 DPT), or any other systemic disease that could affect the eye (like uncontrolled systemic hypertension) and history of ischemic heart disease.

### Ophthalmic examination

For this study, only cases with a complete ophthalmological examination, BCVA by an ETDRS chart, digital 133° color fundus photographs (Clarus 500, Carl Zeiss), and SD-OCT imaging (Spectralis; Heidelberg Engineering, Heidelberg, Germany) were used. Age, DM duration, and hemoglobin A1C (HbA1c) were also obtained for each participant.

The evaluation and quantification of the different layers were carried out using the high-density SDOCT raster volume scan of the macula. The macular volume scan consisted of SD-OCT acquisitions (20° × 20°, 49 HR B-scans, 7 frames per scan), 49 raster horizontal B-scans with 1024 A-scans per B-scan and a depth resolution of 3.9 µm. Additionally, hyperreflective retinal spots (HRS), subretinal detachment (SRD), disorganization of the retinal inner layer (DRIL), and other important changes were assessed.

The peripapillary RNFL thickness was measured using a HS circular scan (3,5 mm diameter), centered in the optic disc, for the neurodegeneration assessment.

### Epigenetic profile

During the routine consultation, 1 mL of blood was collected, and the total RNA was extracted using the Quick-RNA™ Whole Blood (Zymo Research), according to the manufacturer's instructions. The concentration and purity of all RNA samples were determined in NanoDrop One spectrophotometer (Thermo Scientific). One-step NZY RT-qPCR Green kit (NZYtech) was used for first-strand cDNA synthesis and subsequent quantitative real-time PCR (qRT-PCR) in a final volume of 20 μL and performed on the CFX Connect™ Real-Time PCR Detection System to quantify gene expression. Each reaction took place in triplicate using in every reaction non-template controls and specific primers, listed in Table 1, for the genes DNMT1, DNMT3A, DNMT3B and also a reference gene, GAPDH, which was used for data normalization. The cycling conditions were as follows: 50 °C for 15 min; 95 °C for 5 min; and 40 cycles of 95 °C for 15 s and 60 °C for 45 s with fluorescent reading. Then, the relative quantification of the target genes was undertaken by normalizing threshold cycles (Ct) with the mean Ct of GAPDH. Transcript levels were analyzed by calculating ΔΔCt (ΔΔCt = ΔCt treatment − average ΔCt control) and the obtained ΔΔCt values were subsequently log 2-transformed for graphical proposes.

**Table 1.** Primer sequences, accession numbers and product lengths for qRT-PCR analysis.

Genes	Accession Number^a^	Forward primer (5′→ 3′)	Reverse Primer (3′→ 5′)	Product Length (bp)
*GAPDH*	NM_002046.7	GAGTCAACGGATTTGGTCGTA	GCAGAGATGATGACCCTTTTG	245
*DNMT1*	NM_001379.4	CCTCCAAAAACCCAGCCAAC	TCCAGGACCCTGGGGATTTC	101
*DNMT3A*	NM_022552.5	CCAACATCGAATCCATGAAA	CTTGCGCTTGCTGATGTAGT	140
*DNMT3B*	NM_175850.3	CGAATTTTACCACCTGCTGAATT	AGAACGGCCGGTCATCAC	59

^a^ NCBI Reference Sequence (National Center for Biotechnology).

### Statistical analyses

Descriptive statistics was conducted to characterize clinical and demographic parameters, where mean and standard deviation for continuous variables and percentages for categorical variables were used. To compare the three groups, the one-way ANOVA was used when it was possible and Kruskal-Walis test as an alternative. Multiple comparisons were performed using Tukkey test. Results were considered statistically significant when p < 0.05.

All Statistical analysis was performed using R (https://www.r-project.org/) and IBM® SPSS® Statistics V.26.0 software (Armonk, NY, USA).

## Results

The initial 27 eyes/patients recruited were divided into three groups according to the DR presence/absence and DME therapeutic response: CG (n = 11), rDME (n = 9) and nrDME (n = 7). The comparison of demographic and clinical characteristics according to diabetic macular edema response and control group are summarized in Table 2.

**Table 2.** Comparison of clinical characteristics of DME patients and healthy control subjects.

	Control (n = 11)	rDME (n = 9)	nrDME (n = 7)	p-value
Age, years				
Mean (SD) [min.-max.]	74.3 (6.8) [65–85]	69.8 (7.3) [59–80]	69.7 (9.9) [61–90]	0.355^2^
Sex, n (%)				
Female	5 (45.5)	4 (44.4)	6 (85.7)	0.175
Male	6 (54.5)	5 (55.6)	1 (14.3)	
Study eye, n (%)				
Right eye	6 (54.5)	3 (33.3)	4 (57.1)	0.549
Left eye	5 (45.5)	6 (66.7)	3 (42.9)	
BCVA, ETDRS letters				
Mean (SD) [min.-max.]	81.9 (2.6) [77–85]	68.6 (8.8) [58–80]	59 (13.4) [40–75]	**<0** **.** **001^2a^**
Diabetes duration, (years)				
Mean (SD) [min.-max.]	18.3 (6.2) [10–30]	22.2 (10.4) [13–44]	21.7 (10.8) [8–35]	0.575^2^
HbA1c, %				
Mean (SD) [min.-max.]	7.3 (0.7) [6.4–8.5]	8.5 (1.5) [6.8–10.9]	9.6 (2.1) [6–12]	**0** **.** **033^2b^**
Body mass Index (BMI), kg/m^2^				
Mean (SD) [min.-max.]	27.2 (3.6) [23.9–25.9]	29.2 (3.6) [25.7–33.9]	32.3 (5.7) [28.4–43.6]	0.086^1^
Diabetic retinopathy severity (%)				
No or minimal NPDR	11 (100)	1 (11.1)	0 (0)	
Mild NPDR	0 (0)	1 (11.1)	1 (16.7)	n/a
Moderate NPDR	0 (0)	1 (11.1)	1 (16.7)	
Severe NPDR	0 (0)	6 (66.7)	4 (66.7)	
Diabetes treatment, n (%)				
Diet	1 (11.1)	0 (0)	0 (0)	
Oral agents	5 (55.6)	4 (44.4)	0 (0)	n/a
Oral agents + insulin	3 (33.3)	1 (11.1)	5 (71.4)	
Insulin	0 (0)	4 (44.4)	2 (28.6)	

BCVA: best-corrected visual acuity; HbA1C (%) glycated hemoglobin. ^1^DME patients and controls subjects were compared by Kruskal-Wallis. ^2^Group variations between DME patients and controls subjects were evaluated by one-way ANOVA with a post hoc Tukey test. Superscript letters refer to groups that differ at 0.05 level: a = Control Group vs. rDME vs nrDME, and b = Control group vs. nrDME. The bold p-value is statistically significant (p < 0.05).

**Table 3.** Comparison of retinal metrics obtained through SD-OCT between the distinct DME response and control group.

	**Control** **(n = 11)**	**rDME** **(n = 9)**	**nrDME** **(n = 7)**	**p-value**
CRT, µm				
Mean (SD) [min.-max.]	265.5 (16.9) [244–294]	323.3 (70.2) [218–453]	405 (53.9) [343–498]	<0.001^a^
RNFL thickness, µm				
Mean (SD) [min.-max.]	103.5 (10.3) [87–124]	97.8 (10.7) [83–117]	101.3 (8,4) [89–111]	0.461
GCL-IPL thickness, µm				
Mean (SD) [min.-max.]	38.1 (3.1) [33–42]	38.7 (4.3) [29–45]	43.9 (4.5) [37–51]	0.013^bc^
Sub-foveal Choroidal thickness, µm				
Mean (SD) [min.-max.]	268.2 (120) [71–414]	219.9 (73.8) [135–358]	281.6 (71.3) [199–410]	0.388

CRT: Central Retinal thickness; min: minimum; max: maximum. DME patients and controls subjects were evaluated one-way ANOVA with a post hoc Tukey test. Superscript letters refer to groups that differ at 0.05 level: a = Control Group vs. rDME vs nrDME, b = Control group vs. nrDME and c = rDME vs. nrDME.

Concerning the main sociodemographic characteristics, no significant differences were found concerning age (p = 0.355), sex (p = 0.175) and studied eye (p = 0.549).

As expected, BCVA assessment showed a statistically significant difference (p < 0.001) between the studied groups with CG, rDME and nrDME showing 81.9, 68.6 and 59 letters, respectively.

In the metabolic control evaluation, it was observed that despite the significant differences obtained across the studied groups (p = 0.033), these are only verified between the CG and nrDME. With minimum and maximum values very similar between rDME and nrDME groups, no significant HbA1c differences were found through multiple comparisons.

For the remaining clinical characteristics, the CG patients were treated with oral agent (55.6%) or oral agents and insulin combined (33.3%) treatment, the rDME showed mostly oral agents (44.4%) or insulin (44.4%) treatment approach, and the nrDME was mainly treated with oral agents and insulin combined (71.4%) or only insulin (28.6%). Although all CG eyes were classified as “no or minimal NPDR” (100%), rDME eyes were classified as “mild NPDR and severe NPDR” (88.9%) and all nrDME eyes were classified as “mild NPDR and severe NPDR” (100%) no significant differences were found in diabetes duration (p = 0.575). Regarding the Body mass Index (BMI) assessment, despite the nrDME highest values, no significant differences were found between the groups (p = 0.086).

By comparing the different retinal metrics obtained through SD-OCT in Table 3, considering the distinct DME response and CG there are significant differences in CRT (p < 0.001) and GCL-IPL thickness (p = 0.013). The highest CRT value is found in nrDME (405 µm), followed by rDME (323.3 µm) and CG (265.5 µm). The highest GCL-IPL thickness values were obtained in nrDME (43.9 µm), followed by rDME (38.7 µm) and CG (38.1 µm).

No significant differences were found between groups regarding total RNFL thickness (p = 0.461) and Choroidal Subfoveal thickness (p = 0.388).

**Figure 1.**
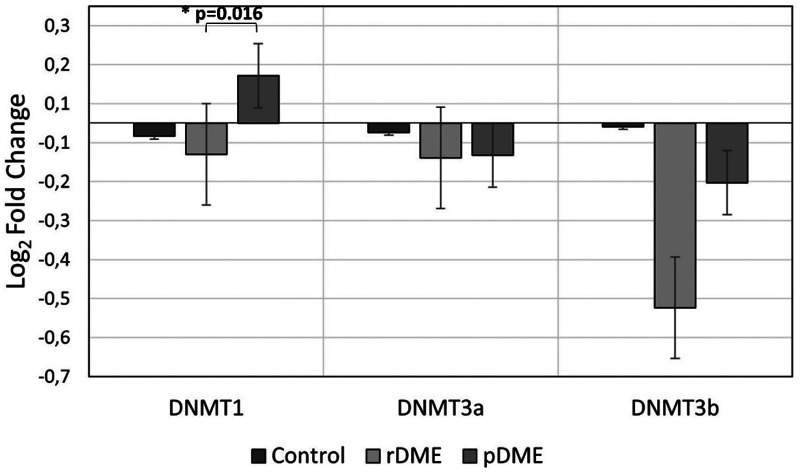
Data represent the relative expression for the DNA methyltransferase genes (DNMT1, DNMT3A and DNMT3B) considering the distinct DME response and control group. GAPDH was used for normalization. (ΔΔCt values were log-transformed). Significant statistical values, which were calculated by one-way ANOVA with a post hoc Tukey test, are illustrated as: *p < 0.05.

Figure 1 shows an evident upregulation in DNMT1 expression (p = 0.016) in nrDME (−0.265+/−0.772) compared to rDME (0.404+/−1.116). Without statistically significant differences (p = 0.123) there is a minor difference in DNMT1 expression in the rDME group compared to the control group (−0.111+/−1.477). Despite this study have not found any statistically significant differences in *de novo* DNMTs (DNMT3A and DNMT3B), a downregulation in DNMT3A expression was observed in the nrDME group (−0.273+/−0.570) when compared with the CG (−0.080+/−0.949), while the rDME group (−1.572+/−1.537) showed an important decrease in DNMT3B expression when compared with the CG (−0.031+/−1.954) and nrDME (−0.507+/−2.256).

On a very preliminary data,Table 4 shows some correlations between the main study parameters. The DNMT1 gene showed a positive correlation (r = 0.617; p = 0.043) with BCVA in CG, a positive correlation (r = 0.917; p = 0.010) with HbA1c in nrDME and a negative correlation (r = −0.659; p = 0.049) with GCL-IPL total thickness in rDME.

**Table 4.** Correlation analysis across the main study parameters examined via Spearman's correlation test.

	DNMT1			DNMT3A			DNMT3B		
	CG	rDME	nrDME	CG	rDME	nrDME	CG	rDME	nrDME
BCVA									
Pearson correlation	**0**.**617**	−0.596	0.125	0.304	−0.259	−0.498	0.348	0.311	−0.555
p value	** 0**.**043**	0.090	0.813	0.363	0.501	0.314	0.294	0.415	0.253
HbA1c									
Pearson correlation	−0.410	0.102	**0**.**917**	−0.286	−0.198	0.609	**−0**.**815**	−0.281	−0.413
p value	0.273	0.848	**0**.**010**	0.455	0.707	0.199	**0**.**007**	0.589	0.416
DM Duration									
Pearson correlation	−0.389	0.076	−0.633	−0.255	0.212	−0.652	0.287	0.368	0.540
p value	0.301	0.846	0.177	0.508	0.584	0.160	0.454	0.330	0.269
RNFL Total									
Pearson correlation	−0.201	−0.304	−0.459	−0.160	−0.211	−0.426	**−0**.**664**	−0.036	0.267
p value	0.553	0.427	0.360	0.638	0.586	0.399	**0**.**026**	0.927	0.609
GCL-IPL total									
Pearson correlation	−0.314	**−0**.**659**	−0.756	0.181	−0.030	−0.517	−0.358	−0.293	0.094
p value	0.346	**0**.**049**	0.082	0.594	0.939	0.293	0.279	0.444	0.860
Sub-foveal Choroidal thickness									
Pearson correlation	0.028	0.354	−0.407	0.265	**0**.**890**	−0.378	0.047	−0.147	−0.406
p value	0.935	0.351	0.423	0.432	**0**.**001**	0.460	0.892	0.706	0.425

RNFL: retinal nerve fiber layer; GCL: Ganglion Cell Layer; IPL: Inner Plexiform Layer. The bold P value is statistically significant (P < 0.05).

Finally, DNMT3A gene showed a positive correlation (r = −0.890; p = 0.001) with Sub-foveal Choroidal thickness in rDME whereas DNMT3b showed a negative correlation (r = −0.815; p = 0.007) with HbA1c and RNFL Total (r = −0.664; p = 0.026) in CG.

## Discussion

In this research, we first demonstrated that metabolic control and disease duration, while important biomarkers^2^ are insufficient to explain the variability of disease progression.^3^ Similar to other studies,^3^ higher HbA1C levels, associated with poorer metabolic control, worse BCVA and more severe stages of the disease were found in the nrDME group. Interestingly, these HbA1C differences were only present between CG and nrDME. Despite the differences in therapeutic response, no clinically significant differences were found between rDME and nrDME.

Further, all the DME patients performed Intravitreal anti-VEGF therapy, the first-line treatment for center-involved DME,^21^ and showed different responses. Based on the reduction in central retinal thickness on SD-OCT^12^ and the BCVA response^19^ at least 180 days after treatment or medical management a careful definition of responder and non-responder profile was performed.^19^ BCVA is an important functional biomarker of therapeutic response^19^ and patients who start treatments with lower BCVA show a tendency for suboptimal response. Similarly, our group with the worst therapeutic response showed lower BCVA.

Quantitative and qualitative assessment through SD-OCT improved the detection and the study of DR^22,23^ and the CRT is a fundamental parameter for the quantification and progression of DME.^19^ Commonly associated with BCVA, and used in different clinical trials, the CRT differences identified (p < 0.001) highlight the difference between the studied groups. Diabetic inner layers involvement, associated with severity and poor prognosis,^3,23^ is present in nrDME (p = 0.013), with GCL-IPL increased thickness compared to the groups studied. No changes at the RNFL level, commonly associated with neurodegeneration,^3^ were found across all groups.

With no significant differences found in the Choroidal Subfoveal assessment, a choroidal thickening was found in nrDME (281.6 µm) compared to rDME (219.9 µm) or CG (268.2 µm) suggesting vascular and inflammatory involvement in nrDME.^3^

It is still unclear why some good metabolic control patients develop DME, the main cause of visual impairment in DR, and others with poor metabolic control do not.^4^ Additionally, real-life data shows that nearly 40% of DME have an inconsistent response^24^ showing a non-responder profile to anti-VEGF intravitreal therapy.^7^ So, it is necessary to understand what external factors can influence gene transcription and individual metabolic response.

To the best of our knowledge, this is the first study that compared groups with different DME responses, SD-OCT structural findings, with DNA methyltransferase transcriptional patterns divergences (genes *DNMT1*, *DNMT3A* and *DNMT3B*). Interestingly, we observed that patients with similar metabolic profile risk factors (metabolic control, disease duration, BMI) showed associated DNA methyltransferase transcriptional expression patterns fitting with the functional (BCVA) and structural changes of the retina (CRT).

Epigenetic research is currently one of the most relevant hot topics due to the reversible characteristics of its mechanisms, plasticity and phenotype impact, which provide new therapeutic targets for retinal diseases.^11–13^ However, despite the fact that some studies focused on gene transcription associated with cellular behavior in response to environmental changes, in animal or in vitro studies,^18,25^ using non-uniform methodological approaches,^26,27^ which addressed primarily the risk of developing DM or DR, epigenetic studies of DME are still scarce. In fact, epigenetic involvement has been previously studied in relevant mechanisms related to DM and DR (inflammation, oxidative stress, apoptosis) that may be important for therapeutic resistance and retinal impairment due to “metabolic memory”.^16–18^

The importance of metabolic memory was shown in a case-control study where Global DNA methylation appears to be modulated during or possibly before the initial DM stages.^18^ This is an important aspect to clarify some differences of metabolic control in the evolution of DR and DME responses. In the same study, they reported a significant increasing trend in global DNA methylation levels related to DR progression (p = 0.006).^18^

Other targeted approaches have also been developed through animal models. In a diet-induced obesity model, Dnmt1 and Dnmt3a mRNA expression, described as an epigenetic mediator of insulin resistance, was elevated but could be significantly reduced after insulin treatment.^28^ The DR severity and inflammatory pathways,^29^ higher levels of total cholesterol and LDL cholesterol^30^ and physical activity^31^ were associated with MTHFR gene promoter hypermethylation.

Here, we found an evident upregulation in *DNMT1* expression in nrDME compared to both CG and rDME. Interestingly, previous studies demonstrated that DNMT activity and *DNMT1* gene expression were significantly increased in both in vitro model and retinal microvasculature from human donors with diabetic retinopathy,^16,32,33^ and DNMT1 overexpression was also described in DR proliferative stages^34^ and DNA methylation differences were related to many gene expression changes, especially oxidative stress and inflammation associated genes.^16^ Data have reported that long-term oxidative stress increases DNMT activity and *DNMT1* gene expression^35^ even in the presence of adequate metabolic control.

Regarding *DNMT1* epigenetic modulator, our data showed a clear upregulation in *DNMT1* transcriptional expression in nrDME compared to both CG and rDME and downregulation in *DNMT1* transcriptional expression in rDME which is suggestive of a lower impact on the clinical situation.

Furthermore, in relation to the studied *de novo* methyltransferase, previous studies demonstrated that diabetes had no effect on the expression of DNMT3A and DNMT3B^32,35^ and their expression may be associated with therapeutic response rather than pathological condition. Showing an increased expression in obesity, and described as acting primarily as a repressor of gene expression, DNMT3A association with lipidic profile and pro-inflammatory signals (e.g. IL-6, LPS) has been unclear.^28^ Here we observed alterations in DNMT3A gene expression patterns, associated with therapeutic response with slight differences between patients who were or are in treatment compared to CG. Most evidently, rDME group showed an important decrease in DNMT3B gene expression compared with the CG and nrDME.

While DMNT1 gene is expressed in human retinal pigment epithelium (RPE) cells and in endothelial cells, and DNMT3A gene is weakly expressed in some inner nuclear layer cells in the adult mouse retina.^33^ In previous studies, the relation of DNMT3B expression seems closer to DNMT1^36–38^ showing different variations across the disease that may be important to help clarify the mechanisms involved.

Here we hypothesize that the structural and functional differences found in patients with different therapeutic responses, despite similar metabolic control and disease duration, might be linked with epigenetics alterations/patterns.

Like others,^16,32,33^ this work showed an evident upregulation in DNMT1 expression in nrDME revealing a possible link between these DNMTs activities with disease severity and structural findings. In addition, the impact of therapeutics seems to be assessed through de novo methyltransferase expression (related with lipidic profile and pro-inflammatory signals),^28^ and the decrease in DNMT3B gene expression found (CG and nrDME) should be considered in future research.

Although exploratory, this study limitation is related to the sample size, to the cross-sectional approach and to the fact that we studied lymphocyte gene expression and not retinal gene expression. It will be important in the future to evaluate the evolution of the disease prospectively, therapeutic influence and vascular (vascular density through OCTA) and neurodegenerative (RNFL) changes.^39^

In the future, a longitudinal approach will be important to clarify if the identified expression differences (1) reflect disease evolution, (2) translate the therapeutic impact, (3) or can help to predict the therapeutic resistance profile. In any case, in the perspective of precision medicine, epigenetic characterization and its relation with non-invasive structural biomarkers like SD-OCT could help clarify the best medicine practice in order to personalize the therapeutic response^12^ and avoid permanent retinal lesions and irreversible blindness.^24^
